# Transcriptomic and Metabolomic Analyses Provide Insights Into an Aberrant Tissue of Tea Plant (*Camellia sinensis*)

**DOI:** 10.3389/fpls.2021.730651

**Published:** 2021-09-13

**Authors:** Ding-Ding Liu, Jun-Ya Wang, Rong-Jin Tang, Jie-Dan Chen, Zhen Liu, Liang Chen, Ming-Zhe Yao, Chun-Lei Ma

**Affiliations:** ^1^Key Laboratory of Tea Biology and Resources Utilization, Ministry of Agriculture and Rural Affairs, Tea Research Institute of the Chinese Academy of Agricultural Sciences, Hangzhou, China; ^2^Tea Research Institute, Hunan Academy of Agricultural Sciences, Changsha, China

**Keywords:** aberrant tissue, *Camellia sinensis*, metabolome, special bud, transcriptome

## Abstract

Tea plant (*Camellia sinensis* (L.) O. Kuntze) is one of the most important economic crops with multiple mutants. Recently, we found a special tea germplasm that has an aberrant tissue on its branches. To figure out whether this aberrant tissue is associated with floral bud (FB) or dormant bud (DB), we performed tissue section, transcriptome sequencing, and metabolomic analysis of these tissues. Longitudinal sections indicated the aberrant tissue internal structure was more like a special bud (SB), but was similar to that of DB. Transcriptome data analysis showed that the number of heterozygous and homozygous SNPs was significantly different in the aberrant tissue compared with FB and DB. Further, by aligning the unmapped sequences of the aberrant tissue to the Non-Redundant Protein Sequences (NR) database, we observed that 36.13% of unmapped sequences were insect sequences, which suggested that the aberrant tissue might be a variation of dormant bud tissue influenced by the interaction of tea plants and insects or pathogens. Metabolomic analysis showed that the differentially expressed metabolites (DEMs) between the aberrant tissue and DB were significantly enriched in the metabolic pathways of biosynthesis of plant hormones and biosynthesis of phenylpropanoids. Subsequently, we analyzed the differentially expressed genes (DEGs) in the above mentioned two tissues, and the results indicated that photosynthetic capacity in the aberrant tissue was reduced, whereas the ethylene, salicylic acid and jasmonic acid signaling pathways were activated. We speculated that exogenous infection induced programmed cell death (PCD) and increased the lignin content in dormant buds of tea plants, leading to the formation of this aberrant tissue. This study advanced our understanding of the interaction between plants and insects or pathogens, providing important clues about biotic stress factors and key genes that lead to mutations and formation of the aberrant tissue.

## Introduction

Tea is a traditional beverage that has been consumed for thousands of years in China. Drinking tea can stimulate neural activity and aid in digestion because tea contains many secondary metabolites beneficial to human health, such as polyphenols, caffeine and theanine (Ye et al., 2003). According to the classification of Section *Thea* (L.) Dyer regarding the genus *Camellia* L. by Chen et al. (2012), *Camellia sinensis* (L.) O. Kuntze, *C. sinensis* var. *assamica* (Masters) Kitamura and *C. sinensis* var. *pubilimba* Chang are used for tea processing. Like other common flowering plants, tea plant is composed of six major organs, including roots, stems, leaves, flowers, fruits, and seeds. However, we found an aberrant tissue, in *C. sinensis* var. *pubilimba* Chang, whose morphological structure is obviously different from the six organs mentioned above. To the best of our knowledge, this is the first time the aberrant tissue has been found in Section *Thea (L.)* Dyer plants (Supplementary Figures 1A–C). In terms of external morphological characteristics, the aberrant tissue appears to combine the characteristics of FB and DB of tea plants. Interestingly, we found that the morphological features of the popular “Yabao tea” in the tea market are similar to the aberrant tissue (Supplementary Figure 1D). However, it was reported that the vast majority of the raw materials of “Yabao tea” did not come from tea plants (Xin, 2013), Hence, this raises very interesting and important questions: What is this aberrant tissue and why does it appear on tea plants (*C. sinensis* (L.) O. Kuntze). After the preliminary analysis, we speculate this aberrant tissue might belong to a certain kind of leafy-bracted galls that had been reported on other plants (Jiang et al., 2019; Man et al., 2019). Plants are sessile and cannot move freely from their habitat, even if they encounter various environmental stresses (Cao et al., 2020). Therefore, plants have developed various adaptive strategies during evolution, with galls being one of the coping strategies that plants use (Shih et al., 2018). Existing studies has showed that plant galls are generated by the interaction of insects, nematodes, fungi, bacteria, and viruses with buds, flowers, leaves, and roots of plants (Takeda et al., 2019). Generally speaking, gall development can be divided into four processes, including secretion of signaling molecules from insects, perception of the signals by plants, plant cell regeneration and differentiation, and organization of gall tissue (Giron et al., 2016). There is experimental evidence that effectors from insect, including auxin, abscisic acid, and other phytohormones and proteins are involved in gall generation (Takeda et al., 2019). Up to now, the structure and features of many galls has been studied, for example, apricot bud gall and phylloxera (Nabity et al., 2013; Li et al., 2021). However, the molecular mechanism of gall development is still unclear.

In the last decade, the next generation sequencing (NGS) and the multi-omics integrated analysis approaches haves been used widely to study the complexity of various biological systems, which has allowed us to describe various biological process in many organisms. In recent years, transcriptome analysis has been reported in some gall, for example, steroid hormones plays a potential role in regulating development of the cup-shaped galls in *Litsea acuminata* leaves (Shih et al., 2018), the floral organ development and procambium differentiation are involved in phylloxera gall development (Schultz et al., 2019), the invasion of phylloxera can induce the expression of genes related to cell wall synthesis and biotic defense signaling in grape leaves (Nabity et al., 2013). In particular, the integrated analysis of gene expression and metabolites provides a good insight into cellular complexity at the molecular level (Agarrwal et al., 2016). However, few studies have used the integrated method to characterize plant galls. In this study, With FB and DB as the control, we performed tissue section observation, transcriptome sequencing and metabolic analysis of aberrant tissues as well as FB and DB as controls, in order to determine the type of aberrant tissue and the molecular mechanism underlying formation of aberrant tissue.

## Materials and Methods

### Plant Materials and Chemicals

The special bud (SB: aberrant tissue), floral bud (FB) and dormant bud (DB) tissues of tea plants were used in this study. All the samples were collected in November 2018 from Rucheng County, Hunan Province, China. Each sample was prepared in three biological replicates for transcriptome sequencing and five biological replicates for metabolite detection, All samples were immediately snap-frozen in liquid nitrogen and stored at −80°C. In addition, “Yabao tea,” purchased from a tea market in Yunnan Province, was dried and used for determination of biochemical components.

### Detection of Biochemical Components Using HPLC

Gallic acid (GA), gallocatechin (GC), epigallocatechin (EGC), catechin (C), caffeine (CAF), epigallocatechin gallate (EGCG), epicatechin (EC), gallocatechin gallate (GCG), and epicatechin gallate (ECG) were analyzed by HPLC (high performance liquid chromatography, Waters e2695). The chromatographic conditions were as follows: injection volume: 10 uL; column: Synergi^TM^ LC (4 um, 4.6 mm × 250 mm); temperature: 35°C; flow rate: 1.23 mL min^–1^; detection: UV-VIS detector at 231 nm. The mobile phases comprised water containing 0.1% v/v formic acid (A) and acetonitrile (B). The gradient elution was as follows: 96% A from 0 to 41 min, 81.3% A at 42 min, and 96% A from 43 to 45 min. The biochemical components were identified by comparing retention times with those obtained using the standard solutions.

### Tissue Section Observation

The SB, FB and DB tissues were sliced longitudinally (<0.1 cm) and stained using the reported method with minor modification (Cao et al., 2020). There were three biological replicates for each tissue sample. The tissue samples were submerged in fixative solution for more than 24 h at 4°C. Firstly, the sections were dehydrated as follows: xylene I solution for 20 min, xylene II solution for 20 min, anhydrous ethanol I solution for 10 min, and anhydrous ethanol II solution for 10 min. This was followed by 95, 90, 80, and 70% v/v ethanol for 5 min each, and finally the sections were washed in distilled water. Secondly, the slices were stained with 1% w/v safranin for 1 h, followed by rinsing off the excess dye with running water. Thirdly, the slices were destained in 50, 70, and 80% v/v alcohol for 1 min each. Fourthly, the sections were stained with 0.5% w/v Fast Green for 1 min, followed by destaining in anhydrous ethanol I solution for 30 s and anhydrous ethanol II solution for 1 min. The sections were then dried in an oven at 60°C until the xylene was transparent for 5 min, and sealed with neutral gum. Finally, the tissue sections were observed under an optical microscope.

### Ultra-High Performance Liquid Chromatography (UHPLC)

The sample extracts were analyzed using an UHPLC-Q-TOF MS/MS system (UHPLC, Agilent 1290 Infinity system). The analytical conditions were as follows: UHPLC column: Waters ACQUITY UPLC BEH Amide column (1.7 um, 2.1 mm × 100 mm); mobile phase A: water, 25 mM ethanol amine and 25 mM aqua ammonia; mobile phase B: acetonitrile; gradient program: 95% B from 0 to 1 min, from 95% B to 65% B between 1 and 14 min, from 65% B to 40% B between 14 and 16 min, maintaining at 40% B from 16 to 18 min, from 40% B to 95% B between 18 and 18.1 min, and maintaining 95% B from 18.1 to 23 min; temperature: 4°C; flow rate: 0.3 mL/min; and injection volume: 2 μL. The effluent was connected to an ESI-triple quadrupole-linear TOF (Q-TOF)-MS. All the tissue samples were mixed to obtain quality control samples so as to ensure the reproducibility of the entire experiment.

### ESI-Q TOF-MS/MS Conditions

The samples were first separated by UHPLC, and then were subjected to mass spectrometry analysis on a Triple TOF 6600 MASS spectrometer. The positive and negative ion modes of ESI were used for detection. The ESI source operation parameters were as follows: ion source gas I (GSI), gas II (GSII) and curtain gas (CUR) were set at 60, 60 and 30 psi, respectively; source temperature: 600°C, IonSpray Voltage Floating (ISVF): ±5500 V; TOF MS scan m/z ranged from 60 to 1000 Da; Product ion scan m/z ranged from 25 to 1000 Da; TOF MS scan accumulation time was set at 0.2 s/spectrum; Product ion scan m/z accumulation time was set at 0.05 s/spectra. Secondary mass spectrometry was obtained by information-dependent acquisition (IDA), and Declustering potential and Collision Energy of two high-sensitivity patterns were used.

### Metabolite Identification and Quantification

The raw data were converted into mzXML format by Proyeo Wizard, and then the XCMS program was used for peak alignment, retention time correction, and peak area extraction. The metabolite structures were identified by matching precise mass number and secondary mass spectrometry. The data were extracted by the XCMS program, and ion peaks with the missing value >50% were deleted from the group. SIMCA-P 14.1 was used for pattern recognition, and multidimensional statistical analysis was performed after data were pretreated with Pareto scaling. Principal component analysis (PCA) was performed with data of 15 samples (three tissues × five biological replicates) to observe differences in metabolite composition among the three tissues (DB, FB and SB). Metabolites with significant differences in content were identified according to the thresholds of VIP (variable importance in projection) ≥1 and fold change ≥2 or ≤0.5. Differentially expressed metabolites (DEMs) were analyzed by KEGG enrichment with Metaboanalyst and MBRole 2.0 software.

### RNA Extraction, Library Construction, and Sequencing

Total RNA was extracted using an RNAprep Pure Plant Kit (Tiangen, Beijing, China). Agarose gel electrophoresis and a NanoDrop microvolume spectrophotometer were used to examine the purity and integrity of the extracted RNA. Transcriptome sequencing was performed on an Illumina Hiseq 150 platform. FastQC was used for the quality evaluation of the sequencing data. Qubit and Agilent 2100 were used to construct nine RNA library (three tissues × three biological replicates) after filtering of the raw data. The filtering standards included adapter sequence removal via Adapter Removal version 2, quality filtering via the sliding window method, and length filtering via removal of sequences shorter than 50 bp (Wang et al., 2020).

### RNA-Seq Read Mapping and Data Processing

High-quality clean reads were mapped to the *C. sinensis* var. *sinensis* genome (Xia et al., 2020) with HISAT2 software to obtain the read count of genes. Gene expression was quantified (FPKM normalization method) using the Cufflink R package. The DEGs in SB vs. DB, SB vs. FB, and DB vs. FB were identified using the DESeq2 package. Differentially expressed genes (DEGs) were identified according to the two thresholds of | log2(FoldChange)| >1 and *p* value <0.01. DEGs were then subjected to the KEGG pathway enrichment analysis and the GO function analysis using the OmicShare Tools and agriGO v2.0 software, respectively (Tian et al., 2017).

### Quantitative Real-Time PCR Validation

To verify the accuracy of transcriptomic data, 30 differentially expressed genes (DEGs) in SB vs. DB were selected for qRT-PCR verification, including DEGs related to plant hormone signal transduction, plant-pathogen interaction, cell wall metabolism, and photosynthesis. Primers were designed using Primer-BLAST on the NCBI website (Supplementary Table 1). RNA was reverse transcribed using a TakaRa RR047A primeScript RT reagent kit according to the manufacturer’s instructions. qRT-PCR was performed on a LightCycler 480 System with a LightCycler 480 SYBR Green I Master. There were three technical replicates for each sample. The qRT-PCR reaction system (10 μL) consisted of 5 μL of SYBR Green Master Mix, 1 μL of cDNA, 0.5 μL each of forward and reverse primer, and 3 μL of sterile water. The qRT-PCR procedure included 10 min of initiation, followed by 40 cycles at 94°C for 10 s, 58°C for 15 s, and 72°C for 12 s. Relative expression levels were calculated using the 2^–ΔΔCt^ method and normalized according to the actin gene of GAPDH.

## Results

### Phenotypic Characterization and Ultrastructure Analysis of Tea Plant Tissues

**FIGURE 1:**
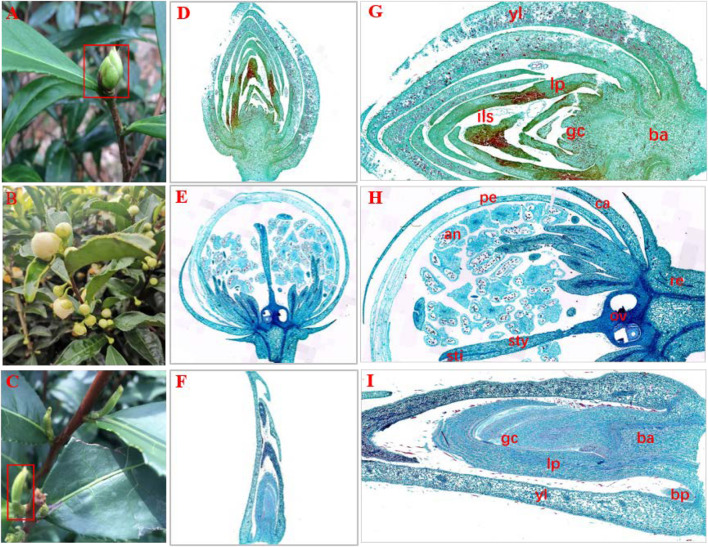
Morphology and histology of flower buds (HB), dormant buds (DB) and an unknown tissue (SB) of tea plants. **(A–C)** Morphology of **(A)** SB, **(B)** FB, and **(C)** DB. **(D–I)** Longitudinal sections of different tissues of SB **(D,G)**, FB **(E,H)**, and DB **(F,I)**. **(G)** ils, insect-like structure. **(H)** pe, petal; an, anther; sti, stigma; sty, style; ov, ovary; ca, calyx; re, receptacle. **(I)** gc, growth cone; yl, young leaves; lp, leaf primordium; bp, bud primordium; ba, bud axis.

In a natural state, SB tissue is apiculate at the top and intumescent in the middle part and base parts, and is similar to tea flower buds (FBs) in shape. In addition, SB is light green and has hard texture (Figure 1A), similar to dormant buds (DBs). Therefore, we initially assumed that the structural features of SB tissues combine the characteristics of FB and DB represent a combination of the characteristics of FB and DB (Figures 1B,C). Further, we observed the tissue sections of SB, FB, and DB to determine morphological features (Figures 1D–F). The longitudinal sections showed that FB and DB had typical internal structures. For example, FBs contained an anther, stigma and ovary (Figure 1H), whereas DBs contained a growth cone and bud primordium (Figure 1I). The internal structure of SB including young leaves, leaf primordium, and growth cone, was totally different from that of FB, but was similar to that of DB. Unlike DB tissue, SB tissue had no bud primordia. Interestingly, we found that the bud axis of SB tissue was significantly intumescent from bottom to top, and whole tissue showed a multi-chamber pattern. Those chambers were composed of many suberized parenchymal cells with numerous insect-like structures distributed in multiple chambers (Figure 1G).

### Identification of SB Tissue in Tea Plants

**FIGURE 2:**
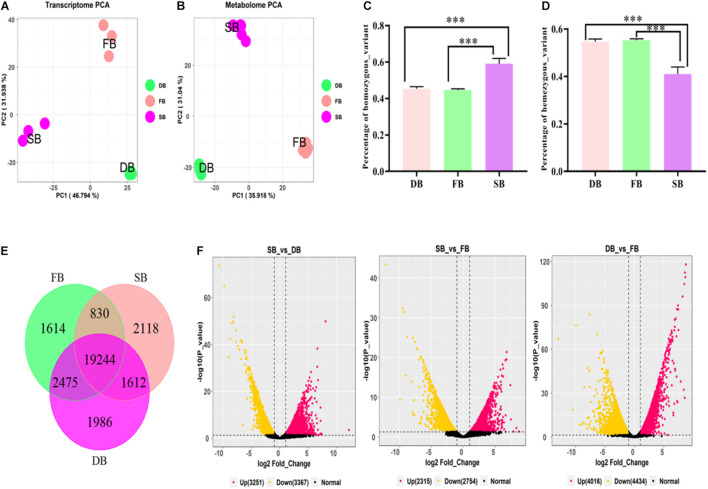
Transcriptomic analysis of SB, FB, and DB tissues of tea plants. **(A)** PCA analysis of gene expression. **(B)** PCA analysis of metabolite content. Statistical analysis of homozygous SNPs **(C)** and heterozygous SNPs **(D)** in SB, FB, and DB. **(E)** Venn diagram of distribution of genes expressed in the three tissues. **(F)** Volcano plots of DEGs in the three tissues.

To reveal whether the aberrant tissue was associated with tea floral buds (FB) or dormant buds (DB), the transcriptomic sequencing and metabolomic analysis of the three tissues were performed. At the transcriptomic level, 41.19–48.88 million clean reads were obtained from the nine RNA-Seq libraries. The percentages of Q30 ranged from 92.88 to 93.88%, indicating that the quality of transcriptome sequencing data was high (Table 1). A total of 50,525 expressed genes were detected in all the samples. The FPKM values of these expressed genes were used for principal component analysis (PCA). The first two principal components explained 78.73% of the total variance (46.79% by PC1 and 31.94% by PC2). The results showed that each sample tissue formed a cluster, and PC1 reflected the remarkable differences among SB, FB, and DB, indicating there were significant differences in gene expression among the three tissues (Figure 2A). At the metabolic level, a total of 10,772 metabolites were detected from the three tissues. The peak areas of those metabolites were used for PCA. The first two principal components explained 66.96% of the total variance (35.92% by PC1 and 31.04% by PC2); the significant differences in metabolites indicated the three tissues were clearly distinguishable (Figure 2B). The PCA results of transcriptome data and metabolome data were consistent.

**TABLE 1:** Quality of transcriptomic data.

Sample	Raw reads	Clean reads	Q30 (%)	Mapped reads
FB_1	44,503,116	41,232,720	93.65	36,398,070 (88.27%)
FB_2	49,213,638	45,503,550	93.51	40,396,218 (88.78%)
FB_3	44,291,502	41,033,710	93.73	36,229,642 (88.29%)
SB_1	42,898,032	39,663,434	93.50	21,558,932 (54.35%)
SB_2	48,381,000	44,633,330	92.88	35,552,160 (79.65%)
SB_3	43,657,617	40,339,550	93.10	32,247,268 (79.94%)
DB_1	41,447,490	38,447,660	93.62	33,852,722 (88.05%)
DB_2	47,615,336	41,071,600	93.88	36,369,764 (88.55%)
DB_3	50,654,558	46,848,556	93.58	41,407,289 (88.39%)

The number of SNPs was analyzed in the three tissues. A total of 85,159 SNPs, including 38,118 homozygous and 47,041 heterozygous SNPs, were detected in FB. A total of 98,228 SNPs comprising 44,802 homozygous and 53,425 heterozygous SNPs were detected in DB. A relatively larger number (128,085 SNPs), including 75,881 homozygous and 42,204 heterozygous SNPs, were detected in SB. The results of ANOVA analysis showed that the numbers of heterozygous and homozygous SNPs in SB were significantly different from those in FB and DB (Figures 2C,D), further indicating that complex mutations occurred in SB tissue.

The tissue-specific expressed genes (TSEGs) and differentially expressed genes (DEGs) in the three tissue types were analyzed. Of the 505,025 expressed genes, those with FPKM ≤0.5 (Cheng et al., 2021) were filtered, and a total of 29,879 expressed genes were obtained, including 23,804 genes expressed in SB tissue, 24,164 genes expressed in FB tissue and 25,318 genes expressed in DB tissue, with many overlapping genes in different tissues. A total of 19,244 commonly expressed genes were observed in the three tissues. In addition, 2,118, 1,614, and 1,986 TSEGs were detected in SB, FB, and DB, respectively (Figure 2E). Our data showed that the number of TSEGs in the SB tissue was remarkably higher than that in FB and DB, and the transcriptional level of TSEGs in the SB tissue was different from that in FB and DB. Subsequently, the DEGs were identified in SB vs. FB, SB vs. DB, and DB vs. FB. A total of 5,069 DEGs were obtained in SB vs. FB, including 2,315 up-regulated and 2,754 down-regulated ones. In SB vs. DB 6618 DEGs were found, with 3,251 up-regulated and 3,367 down-regulated ones. A total of 8,450 DEGs were identified in DB vs. FB, comprising 4,016 up-regulated and 4,434 down-regulated ones (Figure 2F). The number of DEGs was significantly larger in DB vs. FB than in SB vs. FB and SB vs. DB. The difference in the number of DEGs reflected the difference in the function of the tissues. Based on these results, we speculated that SB is a unique tissue distinct from FB and DB.

**FIGURE 3:**
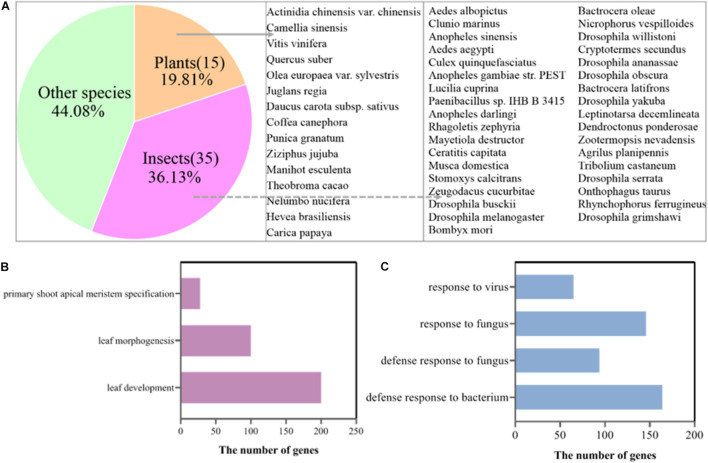
**(A)** Alignment of unmapped sequences of SB tissue to other species sequences. **(B)** GO terms of the genes related to shoot development expressed in the SB tissue. **(C)** GO terms of the pathogen-related genes expressed in the SB tissue.

To confirm this speculation, we mapped the clean reads of the SB, FB, and DB tissues to the tea genome (Xia et al., 2020). A total of 33.85–41.41 million reads of FB and DB were obtained, with alignment efficiency ranging from 88.05 to 88.78%. However, the alignment efficiency of SB only ranged from 54.35 to 79.94%, much lower than that of FB and DB, which might be due to the different tissue type (Table 1). In order to reveal why the SB tissue had a relatively low alignment efficiency, we aligned the unmapped data to the NR database. The results showed many sequences were mapped to insect sequences (Figure 3A). This finding suggested the SB tissue might be a structure produced via the interaction of tea plants and insects or pathogens. To explore this possibility, the Gene Ontology (GO) analysis of 23,804 genes expressed in the SB tissue was performed. The results indicated that some expressed genes were significantly enriched in the GO terms “leaf development,” “leaf morphogenesis,” and “primary shoot apical meristem specification” (*p* ≤ 0.05) that were related to shooting growth and the development of tea plants (Figure 3B). Some expressed genes were enriched in “defense response to bacterium,” “defense response to fungus,” “response to fungus,” and “response to virus” (*p* ≤ 0.05), and these GO terms were related to pathogens (Figure 3C). Combined with the tissue section observations, these findings indicated the SB tissue might be a gall formed via the interaction between dormant buds of tea plants and pathogens or insects.

### Metabolomic Profiling of SB and DB Tissues

**FIGURE 4:**
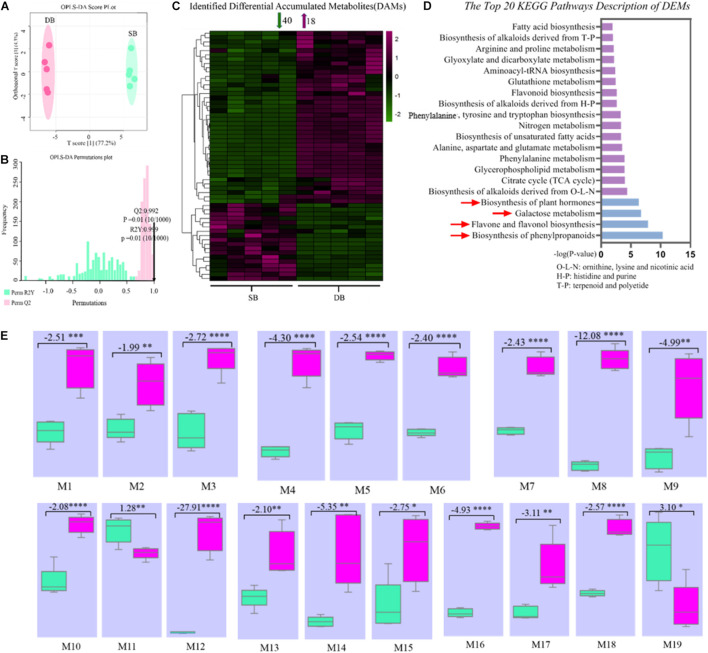
**(A)** OPLS-DA (Orthogonal partial least squares discriminant analysis) score plot of metabolites. **(B)** OPLS-DA permutation plot of metabolites. **(C)** Heat map of identified differentially expressed metabolites. **(D)** KEGG pathway enrichment analysis of metabolites in the SB and DB tissues. **(E)** Box plots of key metabolites in the top four KEGG pathways. (M1-19) Box plots of peak areas of metabolites in the SB and DB tissues. Box plot data represent fold change of metabolites in the SB and DB tissues, with positive values indicating up-regulation and negative values denoting down-regulation. Asterisks indicate the level of significant difference. In panel **(E)**, M1: (-)-epicatechin; M2: 4-hydroxycinnamic acid; M3: alpha-linolenic acid; M4: APIIN; M5: citrate; M6: D-mannose; M7: galactinol; M8: kaempferol; M9: L-arginine; M10: L-glutamate; M11: L-malic acid; M12: quercetin; M13: raffinose; M14: rutin; M15: salicylic acid; M16: shikimate; M17: sinapyl alcohol; M18: sucrose; M19: coniferol. Detailed annotation information on differentially expressed metabolites (DEMs) was shown in Supplementary Table 2. Detailed annotation information on key DEMs was shown in Supplementary Table 3.

In order to further reveal the mechanisms governing formation of SB tissue, we identified 231 metabolites in the SB and DB tissue samples. These metabolites included amino acids, nucleotides, fatty acids, organic acids, derivatives of all aforementioned compounds, flavones and flavone glycosides, and phenylpropanoids (Supplementary Table 1). Subsequently, we screened differentially expressed metabolites (DEMs) using the OPLS-DA multivariate statistical analysis, and the first principal component explained 77.2% of the variation based on the metabolite peak areas in different samples. The results revealed that metabolite composition of the SB and DB tissues was significantly different (Figures 4A,B). we obtained 58 DEMs, including 40 down-regulated and 18 up-regulated DEMs (Figure 4C). Furthermore, these identified DEMs were subjected to the KEGG pathway enrichment analysis. The results indicated that these DEMs were significantly enriched in the biosynthesis of phenylpropanoids, flavone and flavonol biosynthesis, galactose metabolism, and biosynthesis of plant hormones pathways (Figure 4D and Supplementary Table 2). Interestingly, 19 metabolites were enriched in the top four pathways, of which 17 metabolites were significantly downregulated (1.28- to 27.91-fold), including (-)-epicatechin, 4-hydroxycinnamic acid, alpha-linolenic acid, APIIN, citrate, D-mannose, galactinol, kaempferol, L-arginine, L-glutamate, quercetin, raffinose, rutin, salicylic acid, shikimate, sinapyl alcohol, and sucrose (Figure 4E and Supplementary Table 3); most of these DEMs were secondary metabolites, thus indicating an imbalance in the secondary metabolite pathways in the SB tissue.

### Analysis of Metabolic Pathways in SB Tissue

**FIGURE 5:**
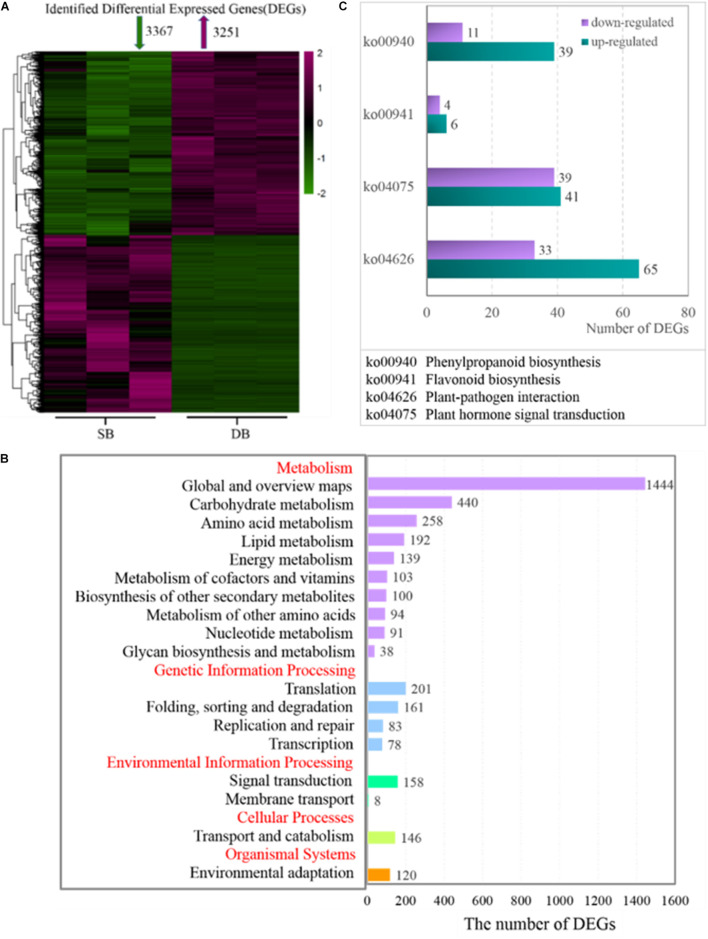
**(A)** Heatmap of identified differentially expressed genes (DEGs) in the SB and DB tissues. **(B)** KEGG pathway enrichment analysis. **(C)** DEG expression in some metabolic pathways. Detailed annotation information of DEGs related to phenylpropanoid biosynthesis, flavonoid biosynthesis, plant-pathogen interaction, and plant hormone signal transduction was shown in Supplementary Table 4.

At the transcriptional level, we further investigated the mechanisms underlying the formation of SB tissue. A total of 6,618 DEGs were screened from the SB and DB groups, including 3,251 significantly up-regulated and 3,367 significantly down-regulated DEGs (Figure 5A). The KEGG enrichment analysis was conducted and the results showed that some DEGs were mainly enriched in the primary metabolism pathways, including carbohydrate metabolism, amino acid metabolism, lipid metabolism, biosynthesis of other secondary metabolites, and energy metabolism (Figure 5B). More importantly, we found that more than half of DEGs were up-regulated in the biosynthesis of other secondary metabolites in the SB tissue. Furthermore, two pathways, plant hormone signal transduction (belonging to signal transduction) and plant-pathogen interactions (belonging to environmental adaptations), were related to biotic stress, and the expressions of genes enriched in these two biotic stress-related pathways changed dramatically in the SB tissue (Figure 5C).

### Analysis of Hypersensitive Response of SB Tissue

**FIGURE 6:**
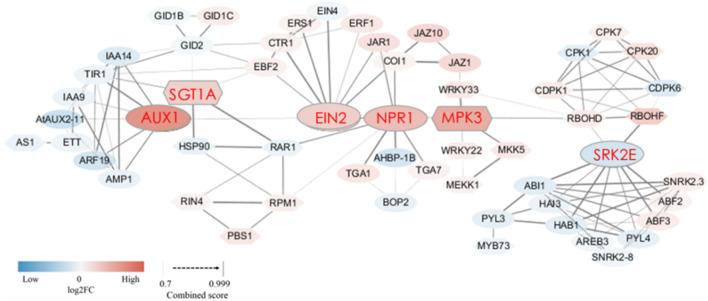
Protein interaction network of DEGs encoding 58 proteins related to plant-pathogen interaction and plant hormone signal transduction in SB_vs_DB. The ovals represent the proteins related to plant hormone signal transduction, and the hexagons represent proteins related to plant-pathogen interaction. The thickness of the connecting line represents the confidence of the predicted protein interaction. The color in each node represents the continuous log2FC. Blue and red colors represent down-regulation and up-regulation, respectively. Detailed annotation information regarding DEGs related to plant-pathogen interaction and plant hormone signal transduction was shown in Supplementary Table 5.

When plants are invaded by pests or pathogens, a series of metabolic pathways such as “plant-pathogen interactions” and “plant hormone signal transduction” produce a variety of defense responses. In this study, we found that the expression of many DEGs related to these two metabolic pathways changed in the SB tissue. To further explore how these metabolic pathways trigger defense responses in the SB tissue, a protein interaction network was constructed (combined score = 0.70–0.99) based on the sequences of DEGs related to “plant hormone signal transduction” and “plant-pathogen interactions.” The network involving phytohormone proteins was related to metabolism of auxin, gibberellin, abscisic acid, salicylic acid, jasmonic acid, ethylene, and proteins related to plant-pathogen interactions. Among the protein-encoding DEGs, 10 DEGs were associated with auxin metabolism, of which nine DEGs were down-regulated. There were three DEGs associated with gibberellin metabolism, of which two DEGs exhibited up-regulation. Six DEGs were involved in ethylene metabolism, of which five were up-regulated. Four DEGs involved in jasmonic acid metabolism were all up-regulated. Four DEGs were related to salicylic acid metabolism, of which two DEGs were up-regulated. In the abscisic acid metabolism pathway, eight DEGs were down-regulated. Furthermore, 15 DEGs in the plant-pathogen interaction pathway were up-regulated in the SB tissue. More importantly, we found some interactive relationships between DEGs through the protein interaction network. Firstly, plant hormone metabolism-related DEGs and plant-pathogen interaction-related DEGs interacted. Among them, SGT1A (the suppressor of the G2 allele of SKP1) was predicted to interact with TIR1 (auxin transport inhibitor response 1, combined score = 0.82), GID2 (F-box protein GID2, combined score = 0.72), and EBF2 (EIN3-binding F-box protein, combined score = 0.75). SGT1A further interacted with RAR1 (cysteine and histidine-rich domain-containing protein, combined score = 0.99) as well as HSP90 (heat shock protein 90, combined score = 0.99). MPK3 (mitogen-activated protein kinase 3) interacted with NPR1 (non-expressor of pathogenesis-related genes 1, combined score = 0.79). JAZ1 (jasmonate ZIM domain-containing protein) interacted with WRKY33 (WRKY transcription factor 33, combined score = 0.77). SRK2E (serine/threonine-protein kinase) interacted with RBOHD (respiratory burst oxidase protein D, combined score = 0.76) as well as RBOHF (respiratory burst oxidase protein F, combined score = 0.98). Various phytohormones also interacted with each other, such as GID2 and TIR1, GID2 and EBF2, EIN2 (ethylene insensitive 2) and NPR1, EIN2 with JAR1 (jasmonic acid-amido synthetase) as well as COI1 (coronatine-insensitive 1), and NPR1 with JAR1 as well as COI1 (Figure 6 and Supplementary Table 5). These results showed that hypersensitive responses (HR) underpinning defense were triggered by the internal interactions between various phytohormone-related genes as well as the interactions between phytohormone-related genes and R/var related genes in the SB tissue.

### Regulation of Cell Wall Metabolism and Secondary Metabolism in SB Tissue

**FIGURE 7:**
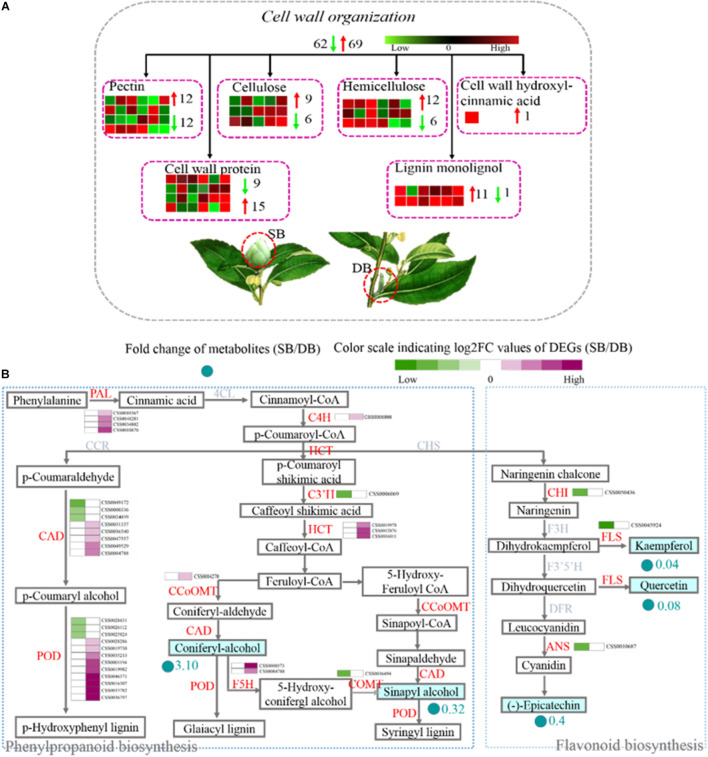
**(A)** Distribution of DEGs related to cell wall metabolism by Blast. **(B)** Expression profile of DEGs related to the phenylpropanoid and flavonoid biosynthesis pathways in SB and DB tissues. PAL, phenylalanine ammonia-lyase; C4H, *trans-*Cinnamate 4-hydroxylase; HCT, shikimate O-hydroxycinnamoyltransferase; CCOMT, caffeoyl-CoA O-methyltransferase; CAD, cinnamyl-alcohol dehydrogenase; CHI, chalcone isomerase; FLS, flavonol synthase; ANS, anthocyanidin synthase; COMT, caffeic acid 3-O-methyltransferase; F5H, ferulate-5-hydroxylase. Detailed annotation information of cell wall metabolism was shown in Supplementary Table 6. Detailed annotation information of DEGs related to phenylpropanoid and flavonoid metabolisms was shown in Supplementary Table 7.

The cell wall is an important part of plant cells. It does not only provide mechanical support, and maintains and determines the morphology of plant cells, but it also acts as a natural barrier to resist various biotic and abiotic stresses during plant growth and development. Pectin, cellulose, hemicellulose, and lignin are the main components of the cell wall. These contents determine the thickness and rigidity of the cell wall. Here, we found that the expression of 131 DEGs related to cell wall metabolism was significantly different in the SB compared with the DB tissue. These DEGs were involved in the metabolic processes (including synthesis, modification, and degradation) of the four cell wall compounds (pectin, cellulose, hemicellulose, and lignin), and they also encoded cell wall proteins and cell wall hydroxycinnamic acid. Among these 131 DEGs, 28 DEGs were involved in the cell wall synthesis, including pectin, hemicellulose, and lignin biosynthesis. Twenty-three out of 28 DEGs exhibited up-regulated expression in the SB tissue, including three DEGs involved in mannan synthesis (CSS0015035: 1.63-fold; CSS0012996: 4.17-fold; and CSS0020384: 2.44-fold) and two DEGs encoding galacturonosyltransferase (CSS0002314: 4.20-fold and CSS0044611: 1.50-fold). In addition, some DEGs related to cell wall extension and expansion were also mainly up-regulated, including two DEGs encoding leucine-rich repeat extension protein (LRX), three DEGs encoding alpha-class expansion protein, and one beta-like-class expansion protein (Figure 7A and Supplementary Table 6). Among the four cell wall compounds, lignin plays an important role in the secondary thickening of cell wall, which can be produced by the “phenylpropanoid biosynthesis” pathway. This pathway also serves as a source of plant secondary metabolites, such as flavonoids. A total of 36 DEGs encoding 12 enzymes were identified, including phenylalanine ammonia-lyase (PAL), *trans*-cinnamate 4-hydroxylase (C4H), peroxidase, ferulate-5-hydroxylase (F5H), shikimate *o*-hydroxycinnamoyltransferase (HCT), caffeoyl-CoA *o*-methyltransferase (CCOMT), 5-*o*-(4-coumaroyl)-D-quinate-3′-monooxygenase (C3’H), cinnamyl alcohol dehydrogenase (CAD), caffeic acid 3-*o*-methyltransferase (COMT), chalcone isomerase (CHI), flavonol synthase (FLS), and anthocyanidin synthase (ANS). Phenylalanine was successively catalyzed by PAL, 4CL, and C4H enzymes to synthesize *p*-coumaroyl-CoA which is the initial substrate for lignin and flavonoid biosynthesis. In the SB tissue, PAL (four DEGs) and C4H (one DEG) genes were all up-regulated (1.39- to 4.24-fold), and HCT (three DEGs), CCOMT (one DEG), F5H (two DEGs), CAD (five DEGs) as well as peroxidase (nine DEGs), all of which were related to lignin synthesis, were also up-regulated (1.05- to 5.85-fold). In contrast, CHI (one DEG), FLS (one DEG) and ANS (one DEG) were all down-regulated (−2.72 to −1.70 times). These results indicated that the upregulation of DEGS related to lignin biosynthesis enhanced lignin biosynthesis and decreased flavonoid metabolism. At the same time, coniferyl alcohol accumulated, whereas sinapyl alcohol content was decreased in the SB tissue. A decrease in sinapyl alcohol content might be related to the down-regulation of the COMT gene. This also indicated that coniferyl alcohol might be the main substrate for lignin biosynthesis (Figure 7B and Supplementary Table 7).

### Regulation of Carbon Metabolism in SB Tissue

**FIGURE 8:**
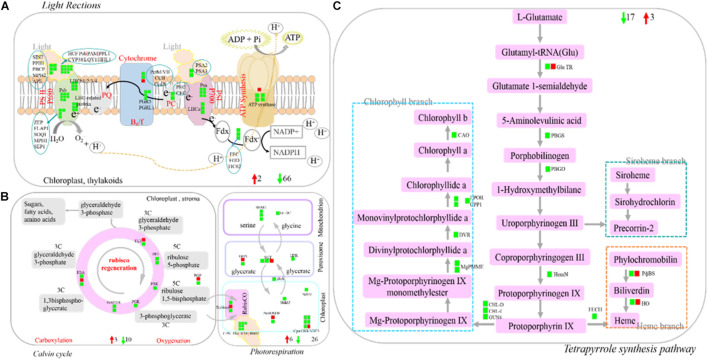
Profile of DEGs involved in photosynthesis and terrapyrrole synthesis pathway. **(A)** DEGs involved in light reaction of photosynthesis. **(B)** DEGs involved in Calvin cycle and photorespiration. **(C)** DEGs involved in Tetrapyrrole synthesis pathway. DEGs were mapped to the photosynthesis diagram according to Log2 fold change (SB/DB). Up-regulated DEGs or down-regulated DEGs were shown in red or green, respectively. Detailed annotation information of DEGs was shown in Supplementary Table 8.

Photosynthesis (light reactions, Calvin cycle, and photorespiration), as one part of carbon metabolism. In this study, 112 photosynthesis-related DEGs were identified, with the vast majority of them being down-regulated. The identified DEGs included light harvesting complex LHCb (−6.76 times), PSII core protein-coding genes (PsbR) (−4.07 times), Psb29 protein (−4.59 times), two early light-induced proteins (ELIPs) (−10.70 to −8.76 times), etc (Figures 8A,B and Supplementary Table 8). Tetrapyrrole synthesis is an important pathway for synthesizing chlorophyll and other photopigments. In the present study, ten DEGs encoding six chlorophyll metabolism-related enzymes were all down-regulated (−1.01 to −4.20 times), such as light-dependent protochlorophyllide oxidoreductase (POR) and chlorophyllide-a oxygenase (CAO) (Figure 8C and Supplementary Table 8).

### Gene Expression Validation via qRT-PCR

To validate the reliability of the transcriptome data, we selected 30 DEGs and analyzed their expression levels in the SB and DB tissue by qRT-PCR. These selected DEGs were involved in plant hormone metabolism, plant-pathogen interactions, cell wall metabolism, and photosynthesis (Supplementary Table 6). The qRT-PCR results showed that the expression patterns of 25 out of 30 DEGs were consistent with the transcriptome sequencing data (Supplementary Figure 2), which confirmed the reliability of our transcriptome data.

## Discussion

### Distinguishing Between the SB Tissue and the Commercial Product of “Yabao Tea”

The main categories of Chinese tea are classified as green, black, Oolong, white, yellow, dark, and reprocessed tea. Generally, the raw materials for these tea products belong to the species and varieties from the sect. *Thea* (L.) Dyer. In recent years, more and more new tea products have appeared in the tea market. One of these commercial tea products called “Yabao tea” (comprising mainly the young shoot buds) has a similar appearance to the SB tissue (Supplementary Figure 1). The processing method of “Yabao tea” is similar to Pu ’er tea, and it has a floral fragrance and sweet taste. However, not all the raw materials of “Yabao tea” come from tea plants, with some materials originating from various sources, such as Theaceae, Moraceae, Lauraceae, and other plants. For example, the raw materials of Hawk tea, as a kind of “Yabao tea,” were found to come from the shoot buds of *Litsea coreana* Levl. var. *lanuginosa* (Xin, 2013). Therefore, to figure out the relationship between “Yabao tea” and the SB tissue, we investigated their chemical composition to determine whether they contained the characteristic biochemical components (GA, GC, EGC, C, CAF, EGCG, EC, GCG, and EGC) of tea plants. The results showed that SB and DB tissues contained the characteristic biochemical characteristics of tea plant, but “Yabao tea” had no such substances (Supplementary Figure 3). Similarly (Tadahiro and Shinsuke, 1985; Tadahiro, 1986) found that *C. sasanqua* of Section non-*Thea (L.)* Dyea lacked these substances by detecting the characteristic biochemical components (EC, C, EGC, ECG, and EGCG) of tea plants in *C. sinensis* and *C*. *sasanqua*. Thus, the HPLC data further verified that our newly discovered tissue comes from the Sect. *Thea* (L.) Dyer, whereas the commercial “Yabao tea” is processed from other plants of the genus *Camellia*.

### Identification of the Structural Type of SB Tissue

In the natural state, the external morphology of SB tissue is not only similar to that of “Yabao tea,” but also to that of the floral buds and dormant buds of tea plants. In addition, SB tissue is also similar in external morphology to the immature floral bud of other plants in the genus *Camellia*, such as *Camellia xifongensis* and *Camellia hupehensis* (Gao et al., 2005). Therefore, to understand the SB tissue type, we characterized the sections of the three tissues using optical microscopy. Previous studies have shown that tissue sectioning is an effective approach for examining microscopic morphological features, organogenesis and differentiation of various plant parts to reveal the formation, evolution, and phylogeny of plant tissues (Lu et al., 2008; Wu et al., 2012). In this study, the main internal structure of SB tissue was found to be similar to that of the DB tissue, but completely different from that of the FB tissue. Young leaves, leaf primordia, and growth cones were shared by the SB and DB tissues (Figures 1H,I), but many insect-like structures were only observed in the SB tissue (Figure 1I). Compared with DB tissue, the SB tissue was enlarged and had many chambers. Jeppson et al. (1975) also found multi-chamber structures in the globular galls produced by *A. phloeocoptes* invading the shoot of plum plants. Based on these results, we speculated the SB tissue might be “galls” formed by the pathogen or insect invasion into tea shoot buds. To verify this speculation, transcriptomic sequencing and metabolomic analysis were further performed in the three tissues. A total of 50,525 expressed genes and 10,772 metabolites were obtained in SB, FB, and DB. The PCA plot of the expressed genes and the metabolites showed the three tissue samples were clearly separated. In addition, the analysis of SNP numbers, TSEGs, and DEGs in the three tissues indicated the SB tissue might have more complex mutations than the FB and DB tissues. The DEG transcription levels in SB were different from those in the FB and DB tissues. The results of the alignment of the sequencing data from the three tissues against the tea reference genome showed the FB and DB tissues had 88.05–88.78% mapping rates, whereas the SB tissue only had 54.35–79.94% mapping rates. The unmapped reads of the SB tissue were aligned to the NR database, and 36.13% sequences were mapped to insect sequences. In addition, a total of 328 genes expressed in the SB tissue were significantly enriched in the GO terms leaf development, leaf morphogenesis and primary shoot apical meristem specification (*p* ≤ 0.05), all of which were related to shoot growth and development. For example, the *CSS0018865* gene encoding NAC transcription factor was expressed highly in SB and DB, but barely at all in FB. Studies haves shown that the expression of the NAC gene (DRL1) decreased with advancing leaf senescence in grapevine, but overexpressing the DRL1 gene significantly delayed leaf senescence in tobacco plants (Zhu et al., 2019). A total of 469 expressed genes were also mapped to the pathogen-related GO terms defense response to bacterium, defense response to fungus, response to fungus, and response to virus (*p* ≤ 0.05). For example, the *CSS0008271* gene encoding mitogen-activated protein kinase 3 was highly expressed highly in SB, but little in FB and DB. For example, Han et al. (2010) found that Mitogen-activated protein kinases 3 and 6 regulate *Botrytis cinerea*-induced ethylene production in Arabidopsis. Hence, these results indicated the SB tissue might be a gall tissue formed from the interaction of dormant buds of tea plant and pathogens or insects.

### Characterizing the Mechanisms Governing Generation of SB Tissue

As a plant tissue, SB will inevitably produce complex defense mechanisms in response to various environmental stresses, including biotic (insects or pathogens) or abiotic stresses. As a local resistance reaction, a HR can quickly kill the cells near the infection site, deprive the pathogens or insects of nutrients, and prevent infection spread. The plant cell death caused by defensive HR is called PCD (Novák et al., 2013; Heath, 2000). Some studies have reported that phytohormones, ROS (reactive oxygen species), NO (nitrogen oxide), and other signaling molecules promote PCD during the hypersensitive response (HR) (He et al., 1996; Koch et al., 2000; Raffaele et al., 2006; Senthil-Kumar and Mysore, 2012; Vandelle et al., 2016). In this study, we constructed a predicted protein interaction network, in which some DEGs encoded the proteins related to plant hormone signal transduction, and other DEGs encoded proteins related to plant-pathogen interaction. We found that DEGs related to auxin and abscisic acid were mainly down-regulated, and that DEGs related to ethylene, jasmonic acid and salicylic acid were mainly up-regulated in the SB tissue. Ciaghi et al. (2019) also found that, compared with clubroots, the jasmonic acid synthesis genes were down-regulated in symptomless roots of *Brassica oleracea* var. *gongylodes*. The predicted protein interaction network showed that the SGT1, RBOHD, and RBOHF proteins were involved in a relatively complicated network, and they interacted with some proteins related to plant hormone signal transduction. The up-regulation of genes encoding the above-mentioned three proteins directly affected the HR in the plant-pathogen interaction pathway (Figure 6), suggesting that phytohormones affected the HR and led to PCD in the SB tissue. Previous studies have reported that interactions among SGT1, RAR1, SCF (Skp1-Cullin-F-box, ubiquitin ligase complex), and COP9 (signalosome subunits) in barley leaves indicated a link between disease resistance and ubiquitination. TIR (Transport inhibitor response 1) is an F-box protein related to auxin, and auxin causes the SCF^*TIR*1^ complex to target negative regulators of the ubiquitin-mediated degradation pathway (Gray et al., 2001; Azevedo et al., 2002; Tan et al., 2007; Yabuta et al., 2019). A yeast two-hybrid assay showed that GID2, also acting as an F-box protein, interacted with the rice SKP1 homolog protein, and that GID2 positively regulated ubiquitin-mediated degradation of SLR1 (gibberellin (GA) signal inhibitor) by the SCF^*GID*2^-proteasome pathway (Sasaki et al., 2003). Molecular characterization revealed that the functions of EBF2 (EIN3-binding F-box 2) were similar to those of TIR1 and GID2. In the absence of ethylene, EIN2 activated the transcription factor EIN3, and EIN3 is quickly degraded by the EBF1/EBF2-mediated ubiquitin-proteasome (Choi et al., 2014; Vidhyasekaran, 2014). Our data showed that in the SB tissue, SGT1A (as an important hub protein) interacted with the TIR1, GID2, and EBF2 proteins, that the genes encoding TIR1 and GID2 proteins were down-regulated and the gene encoding EBF2 protein was up-regulated; more importantly, EIN2 was also up-regulated as a gene upstream of EBF2. A previous study has reported that ethylene biosynthesis is often significantly increased during infection by pathogens (Vidhyasekaran, 2014). After comparing the resistance of the auxin and gibberellin signal transduction pathways against pathogens or insects, we proposed that the ethylene signal transduction pathway might play a major role in the tea plant responses to biotic stress. Furthermore, we also found the EIN2 protein interacted with the JAR1 and NPR1 proteins, and the genes encoding these two proteins were both up-regulated in the SB tissue. In *Arabidopsis thaliana*, the susceptibility degree of the *ein2*, *jar1*, *npr1* triple mutants to *B. cinerea* was significantly higher than that of wild-type and the *ein2, jar1*, and *npr1* single mutants (Ferrari et al., 2003). These findings provided further evidence that ethylene signal transduction pathway together with the jasmonic acid and salicylic acid signaling is involved in defense responses.

In the present study, we found the SB tissue had hard texture, which might be related to its cell walls. We hypothesized that the SB tissue might respond to environmental stresses through a cell wall reinforcement mechanism. The most visible role of the plant cell wall is to determine the cell size and shape (Taiz, 1984). Plant cell walls have thickness and rigidity, and they provide mechanical support for plant. Another important function of the cell walls is to act as a natural barrier against invasion by pathogens or attack by herbivorous insects during plant growth and development (Lagaert et al., 2009; Underwood, 2012). The typical plant cell wall consists of the primary and the secondary cell wall, and the middle lamella. The primary cell wall is composed mainly of cellulose, hemicellulose, and pectin, whereas the secondary cell wall contains mainly cellulose, hemicellulose, and lignin (Wang and Dixon, 2012; Kumar et al., 2016; Liu et al., 2021). In addition to polysaccharides and lignin, proteins are also the major components of the cell wall.

In the present study, we found that many DEGs related to pectin, hemicellulose, and lignin synthesis were up-regulated in the SB tissue. Interestingly, except for C3’H gene, almost all the lignin synthesis-related DEGs were up-regulated (Figure 7A and Supplementary Table 6). Coumaryl alcohol, coniferyl alcohol, and sinapyl alcohol are the three monolignols essential in plant lignin synthesis, and they are synthesized by the phenylpropanoid biosynthetic pathway (Tobimatsu et al., 2013). Our metabolomic data indicated coniferyl alcohol content was significantly increased (3.10-fold), but sinapyl alcohol content was obviously decreased (0.32-fold) in the SB tissue. Our transcriptomic data showed that 3 PAL, 1 C4H, 1 HCT, 5CAD, 1 CCOMT, 9 Peroxidases, and 2 F5H were all up-regulated, whereas 1 COMT was down-regulated in the SB tissue. Among them, C4H enzyme acts upstream of the phenylpropanoid biosynthetic pathway and influences the monolignol content in transgenic alfalfa and tobacco; moreover, the down-regulation of C4H gene has been reported to significantly decrease lignin content (Sewalt et al., 1997; Reddy et al., 2005; Chen et al., 2006). HCT enzyme acts in the mid-stream of the phenylpropanoid biosynthetic pathway, and HCT deficiency leads to lignin content reduction, flavonoid hyperaccumulation, and growth inhibition in *Arabidopsis* (Li et al., 2010; Pasold et al., 2011). CAD and COMT enzymes act downstream of the phenylpropanoid biosynthetic pathway, and CAD enzyme catalyzes *p*-coumaraldehyde, coniferyl aldehyde, and sinapaldehyde into *p*-coumaryl alcohol, coniferyl alcohol, and sinapyl alcohol monolignols, respectively (Pedersen et al., 2005; Day et al., 2009; Vanholme et al., 2010). COMT enzyme also catalyzes the formation of coniferyl alcohol and sinapyl alcohol. Subsequently, these monolignols are polymerized by peroxidases to form lignin (Xia et al., 2018). In *Arabidopisis* and alfalfa, the mutation of the genes encoding CAD, COMT, and peroxidases has been reported to result in lignin content reduction (Guo et al., 2001; Siboout et al., 2005; Shigeto et al., 2015). Taken together, the down-regulation of all the above-mentioned genes has led to the reduction of lignin levels in various species. In contrast, we found that the C4H, HCT, and CAD genes were up-regulated, which was consistent with the accumulation of coniferyl alcohol in the SB tissue. A decrease in sinapyl alcohol content may have been related to the down-regulation of the COMT gene. Based on the above results, we speculated that guaiacyl lignin might be the main lignin type deposited in the cell walls of SB tissue. Furthermore, it has been reported that the ET signal-mediated monolignol synthesis strengthens the cell wall, thus enhancing *Arabidopsis* plant resistance against *B. cinerea* (Lloyd et al., 2011). In the present study, the genes related to ethylene signal transduction such as EBF2 and EIN2 were up-regulated, together with the genes involved in lignin synthesis. Therefore, we speculated that the ethylene signal might mediate monolignol synthesis in the SB tissue, thus promoting cell wall reinforcement in response to adverse environmental conditions.

Photosynthesis is an important source of energy and carbon skeleton for plant growth and development. Under biotic stress, plants lose photosynthetic capacity and reduce the content of chlorophyll and other pigments in their tissues (Huang et al., 2015). In the present study, almost all the genes involved in photosynthesis were down-regulated, including genes related to light reactions, Calvin cycle and photorespiration, together with the genes involved in the tetrapyrrole synthesis pathway, suggesting the reduced photosynthetic capacity of SB tissue. Similar observations were reported in grape, rice and *L. acuminata*. For example, Nabity et al. (2013) reported that the expression of photosynthesis-related genes decreased in galls compared to that of undamaged tissue. Shih et al. (2018) also reported that the expression of photosynthesis-associated (light and dark reaction) genes was suppressed in cup-shaped galls and their host leaves of *L. acuminata*. In addition, Agarrwal et al. (2016) found that the expression of genes involved in the Calvin cycle and chlorophyll branch of TSP was down-regulated in the infected compared with non-infected tissues. In the present study, citrate, D-mannose, and sucrose (as carbon-containing compounds) participated in carbon metabolism, and their contents were decreased in the SB tissue. These results further confirmed that the photosynthetic capacity of SB tissues declined under stress conditions.

## Conclusion

We revealed a molecular mechanism of formation of an aberrant tissue of tea plants by an integrated analysis of transcriptome and metabolome. Our results suggested the aberrant tissue might be a variation of dormant bud tissue produced via the interaction of tea plants and insects or pathogens; in addition, the exogenous infection might have induced programmed cell death and increased lignin content in dormant buds, leading to the formation of this aberrant tissue (Supplementary Figure 4).

## Data Availability Statement

The RNA-Seq data can be found in GenBank under bioproject number PRJNA753669.

## Author Contributions

D-DL performed the data analysis, interpreted the results, and drafted the manuscript. R-JT performed the experiments. J-DC, LC, and M-ZY gave guidance on the experimental design. J-YW, ZL, and C-LM gathered samples. C-LM planned and designed the research. All authors read and approved the final manuscript.

## Conflict of Interest

The authors declare that the research was conducted in the absence of any commercial or financial relationships that could be construed as a potential conflict of interest.

## Publisher’s Note

All claims expressed in this article are solely those of the authors and do not necessarily represent those of their affiliated organizations, or those of the publisher, the editors and the reviewers. Any product that may be evaluated in this article, or claim that may be made by its manufacturer, is not guaranteed or endorsed by the publisher.

## Supplementary Material

Supplementary Figure 1 Phenotype of SB tissue and “Yabao tea.” **(A–C)** SB tissue **(D)** “Yabao tea.”

Supplementary Figure 2 qRT-PCR validation of DEGs in SB_vs_DB. Error bars above the qRT-PCR columns represent relative ± standard deviation. **(A)** DEGs related to plant-pathogen interaction and plant hormone signal transduction. **(B)** DEGs related to cell wall metabolism. **(C)** DEGs related to photosynthesis. Detailed annotation information on these DEGs was shown in Supplementary Table 9.

Supplementary Figure 3 Chromatograms of biochemical components detected in different samples.

Supplementary Figure 4 Schematic diagram of formation mechanism of SB tissue.

Supplementary Table 1 Information of all metabolites.

Supplementary Table 2 Information of DEMs between SB and DB tissue.

Supplementary Table 3 Annotation information of 19 key DEMs.

Supplementary Table 4 Annotation information of DEGs related to phenylpropanoid biosynthesis, flavonoid biosynthesis, plant-pathogen interaction, and plant hormone signal transduction in SB_vs_DB.

Supplementary Table 5 Data of protein interaction network of DEGs encoding 58 proteins related to plant-pathogen interaction and plant hormone signal transduction in SB_vs_DB.

Supplementary Table 6 Annotation information of DEGs related to cell wall metabolism in SB_vs_DB.

Supplementary Table 7 Annotation information of DEGs related to phenylpropanoid and flavonoid metabolisms in SB_vs_DB.

Supplementary Table 8 Annotation information of DEGs related to photosynthesis and tetrapyrrole synthesis in SB_vs_DB.

Supplementary Table 9 Information of primers used for the qPCR assay.
