# Supplementary figures and images for: Transcriptomic and Metabolomic Analyses Provide Insights Into an Aberrant Tissue of Tea Plant (Camellia sinensis)
Source: Front Plant Sci. 2021 Sep 13;12:730651. doi: 10.3389/fpls.2021.730651 (PMC8474014; doi:10.3389/fpls.2021.730651)

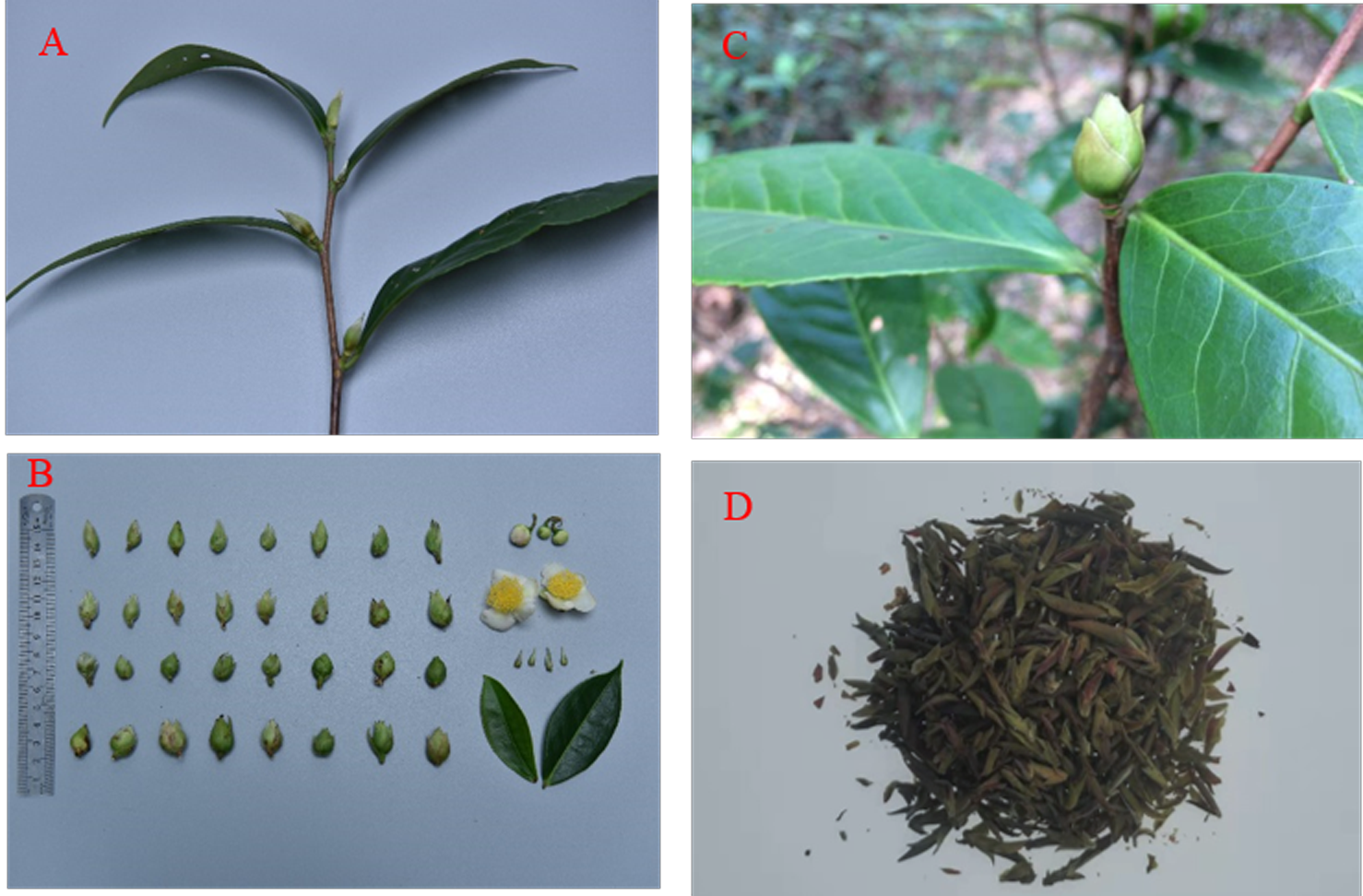

Supplement: Supplementary Figure 1 — Phenotype of SB tissue and “Yabao tea.” (A–C) SB tissue (D) “Yabao tea.” [file Image_1.tif]

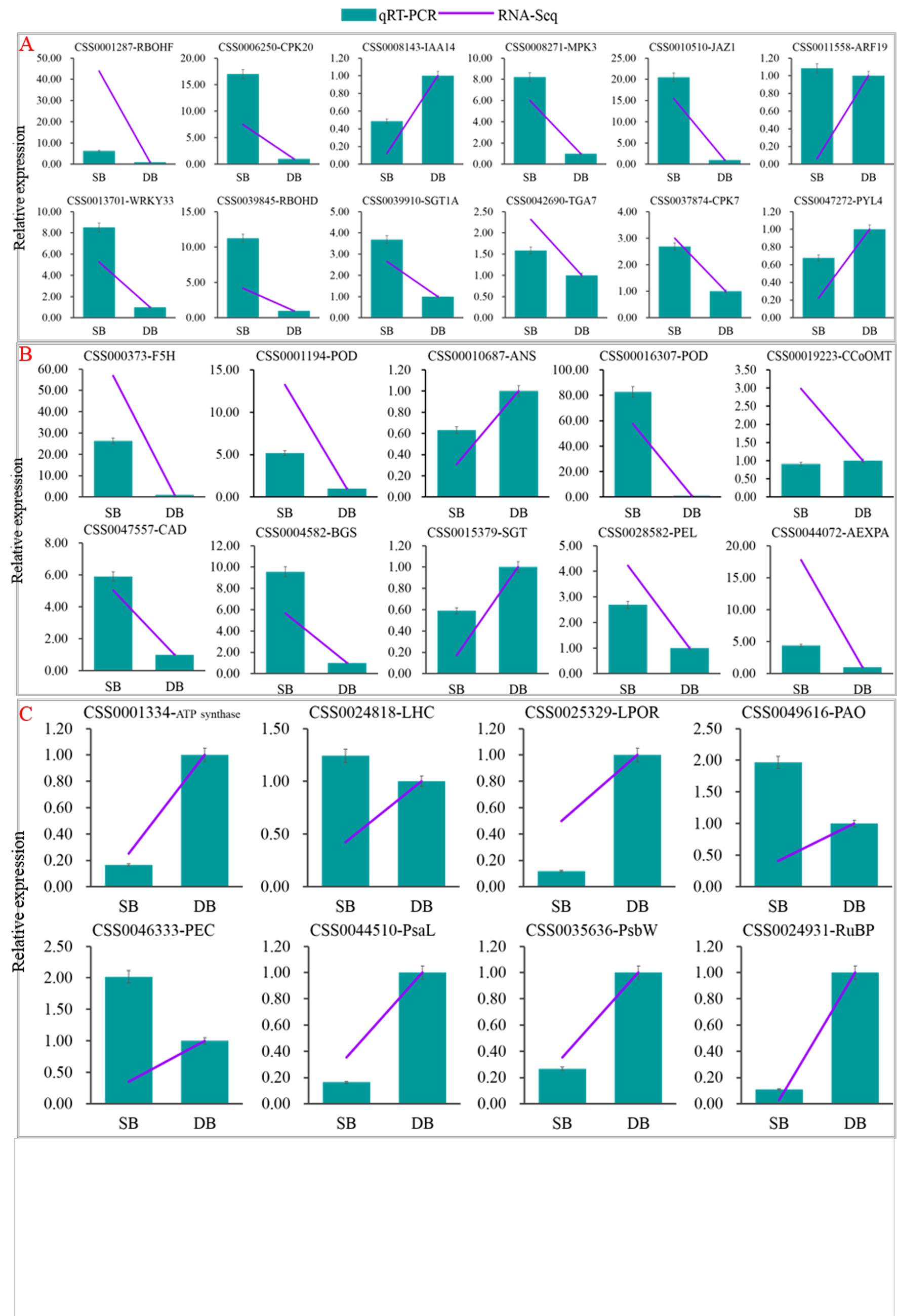

Supplement: Supplementary Figure 2 — qRT-PCR validation of DEGs in SB_vs_DB. Error bars above the qRT-PCR columns represent relative ± standard deviation. (A) DEGs related to plant-pathogen interaction and plant hormone signal transduction. (B) DEGs related to cell wall metabolism. (C) DEGs related to photosynthesis. Detailed annotation information on these DEGs was shown in Supplementary Table 9. [file Image_2.tif]

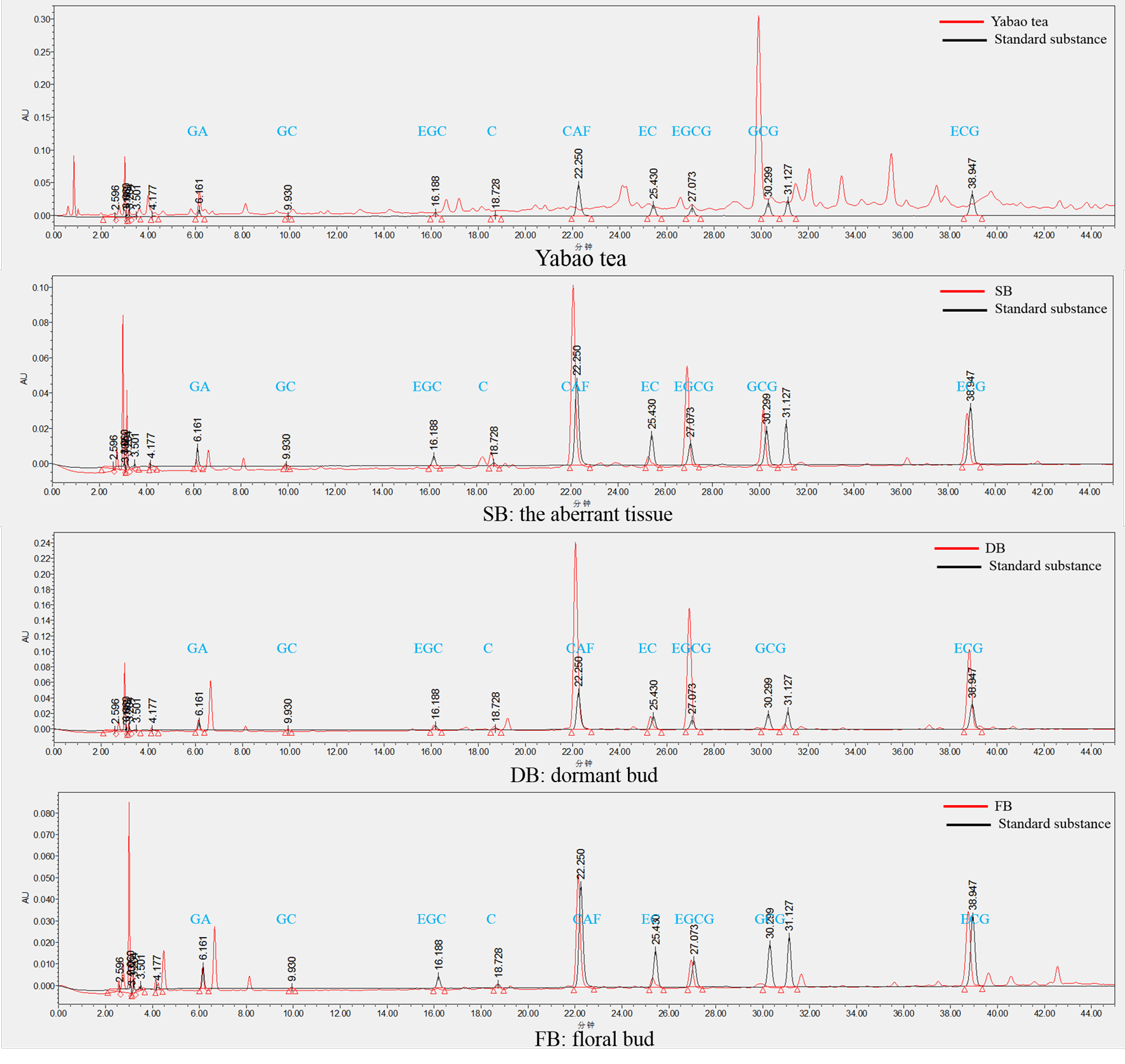

Supplement: Supplementary Figure 3 — Chromatograms of biochemical components detected in different samples. [file Image_3.tif]

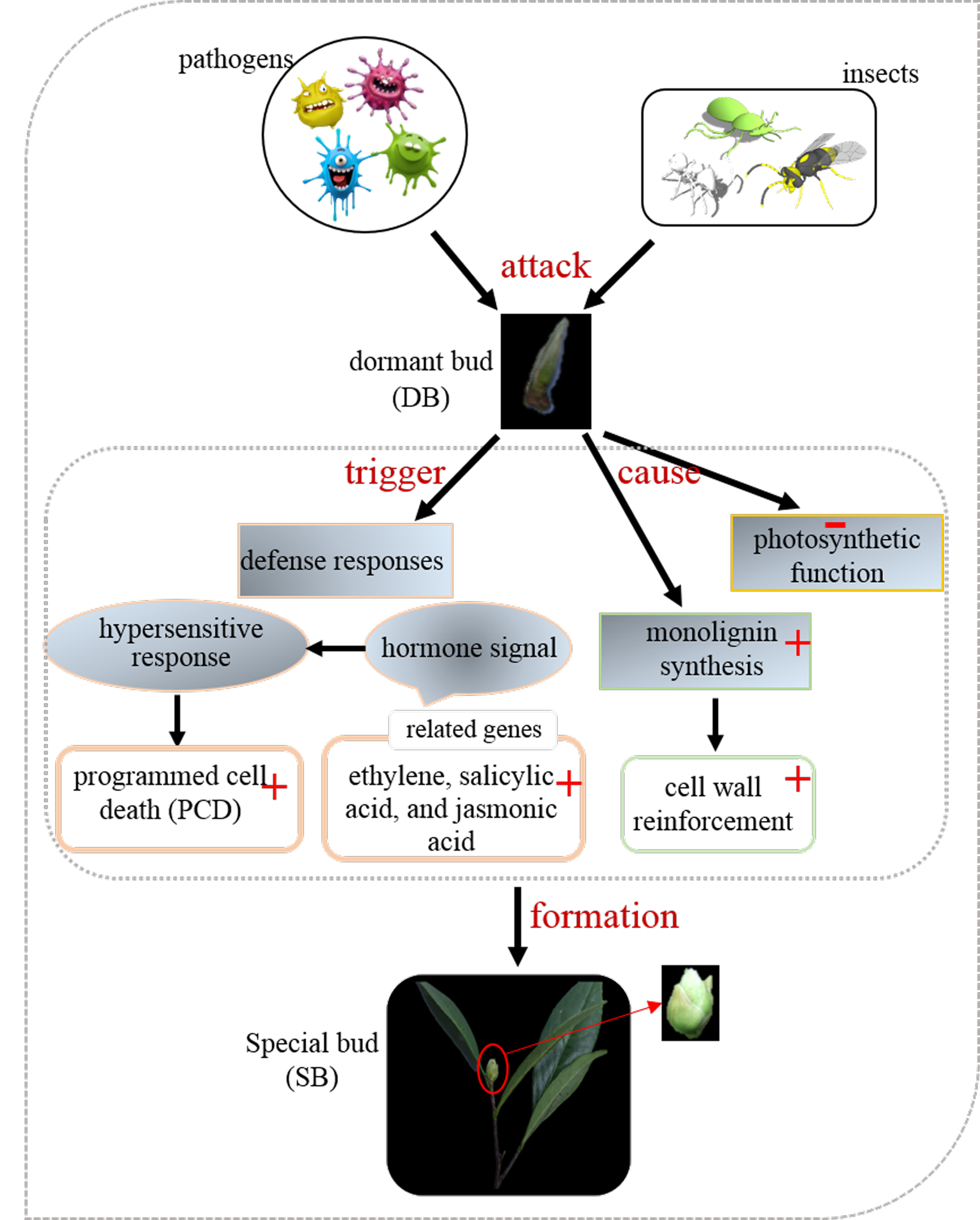

Supplement: Supplementary Figure 4 — Schematic diagram of formation mechanism of SB tissue. [file Image_4.tif]
